# Hsa_circ_0004831 downregulation is partially responsible for atorvastatinalleviated human umbilical vein endothelial cell injuries induced by ox-LDL through targeting the miR-182-5p/CXCL12 axis

**DOI:** 10.1186/s12872-021-01998-4

**Published:** 2021-05-01

**Authors:** Gang Su, Guangli Sun, Jian Lv, Weiwei Zhang, Hai Liu, Yajing Tang, Haoang Su

**Affiliations:** 1 Department of Cardiovascular Surgery, The First Affiliated Hospital of Zhengzhou University, No. 1 Jianshe East Road, Erqi District, Zhengzhou, 453100 China; 2 Department of Ophthalmology, The First Affiliated Hospital of Zhengzhou University, No. 1 Jianshe East Road, Erqi District, Zhengzhou, 453100 China; 3 The Second School of Clinical Medicine, Henan University of Traditional Chinese Medicine, Zhengzhou, China

**Keywords:** Atorvastatin, Ox-LDL, HUVEC, Hsa_circ_0004831, MiR-182-5p, CXCL12

## Abstract

**Background:**

The dysfunction and injury of human umbilical vein endothelial cells (HUVECs) are key events of atherosclerosis (AS). Atorvastatin (ATV) has been shown to play a protective role on endothelial cells. However, the associated molecular mechanisms remain not fully illustrated.

**Methods:**

HUVECs were treated with oxidized low-density lipoprotein (ox-LDL) to mimic the pathological conditions of endothelial cell injury in AS. Cell injuries were assessed according to cell viability, cell apoptosis, cycle progression, oxidative stress and inflammatory responses using CCK-8 assay, flow cytometry assay or commercial kits. The expression of hsa_circ_0004831, miR-182-5p, and C-X-C motif chemokine 12 (CXCL12) mRNA was examined using quantitative real-time PCR (qPCR). The expression of CXCL12 protein was quantitated by western blot. The predicted target relationship between miR-182-5p and hsa_circ_0004831 or CXCL12 was verified by pull-down assay, dual-luciferase reporter assay or RIP assay.

**Results:**

The expression of hsa_circ_0004831 was upregulated by ox-LDL but downregulated by ATV in HUVECs. ATV promoted cell viability and cell cycle progression but inhibited apoptosis, oxidative stress and inflammation in ox-LDL-treated HUVECs, while the role of ATV was partially reversed by hsa_circ_0004831 overexpression. MiR-182-5p was targeted by hsa_circ_0004831, and hsa_circ_0004831 overexpression-restored apoptosis, oxidative stress and inflammation were blocked by miR-182-5p restoration. Further, CXCL12 was targeted by miR-182-5p, and miR-182-5p inhibition-stimulated apoptosis, oxidative stress and inflammation were lessened by CXCL12 knockdown.

**Conclusion:**

Hsa_circ_0004831-targeted miR-182-5p/CXCL12 regulatory network is one of the pathways by which ATV protects against ox-LDL-induced endothelial injuries.

## Highlights

1. Atorvastatin alleviates ox-LDL-induced apoptosis, oxidative stress and inflammation in HUVECs.
2. The expression of hsa_circ_0004831 is upregulated by ox-LDL but impaired by atorvastatin in HUVECs.
3. Atorvastatin ameliorates ox-LDL-induced HUVEC injuries by decreasing the expression of hsa_circ_0004831.
4. Hsa_circ_0004831 recovers ox-LDL-induced HUVEC injuries by targeting the downstream miR-182-5p/CXCL12 axis.

## Background

Atherosclerosis (AS) remains an important public health problem with high morbidity and mortality worldwide, especially in Western developed countries [1]. The pathogenesis and treatment of the disease have been better understood, and growing evidence indicates that endothelial cell dysfunction is an important cause of AS [2, 3]. Risk factors for this disorder, such as smoking, hypertension, diabetes, and sedentary lifestyles can trigger oxidative stress, which is the most important factor in endothelial dysfunction [4]. Besides, monocytes are activated and recruited to the artery wall and then differentiate to macrophages to engulf lipids, thus forming foam cells [5]. During this period, a substantial number of chemokines, cytokines, adhesion molecules, and other proinflammatory factors are activated, triggering inflammatory responses [4, 6, 7]. Oxidized low-density lipoprotein (ox-LDL) has been reported to play a major role in atherosclerosis initiation and development by acting on multiple cells, such as endothelial cells [8], leading to activated pathways of apoptosis, increased reactive oxygen species (ROS) and endothelial dysfunction [8]. The studies on the inhibition of ox-LDL-induced endothelial injuries are urgent to provide additional treatment options for AS.

Atorvastatin (ATV) is a common agent for primary hypercholesterolemia. Clinical trials of ATV have shown a significant reduction in cardiovascular events in patients [9]. Human umbilical vein endothelial cells (HUVECs) have been regarded as a standard model for endothelial cells in healthy or diseased conditions [4]. It was reported that angiotensin II induced several cellular dysfunctions and injuries in HUVECs, including oxidative stress, inflammation, apoptosis and mitochondrial damage, while the usage of ATV alleviated these effects to protect HUVEC function [10]. Besides, ATV was also shown to functionally maintain the stability of lysosomes and mitochondria, thereby inhibiting angiotensin II-induced apoptosis of HUVECs [11]. These findings indicate that ATV plays a vital role in suppressing HUVEC dysfunction. However, the mechanisms of ATV function in HUVEC dysfunctions in AS are not fully uncovered.

Accumulating studies have reported that non-coding RNAs are involved in the progression and pathogenesis of AS [12, 13]. For instance, hsa_circ_0003575 was upregulated in ox-LDL-treated HUVECs, and hsa_circ_0003575 depletion promoted HUVEC proliferation and angiogenesis that were impaired by ox-LDL [14]. Compared to lncRNAs and miRNAs, the functions of circRNAs are still lacking. However, the high stability of circRNAs due to its closed-loop structure without 3′ and 5′ ends makes circRNAs more promising biomarkers for the diagnosis and treatment of diverse diseases and cancers [15]. Therefore, it is urgent to explore the function of specific circRNAs to provide biomarkers for AS.

**Fig. 1:**
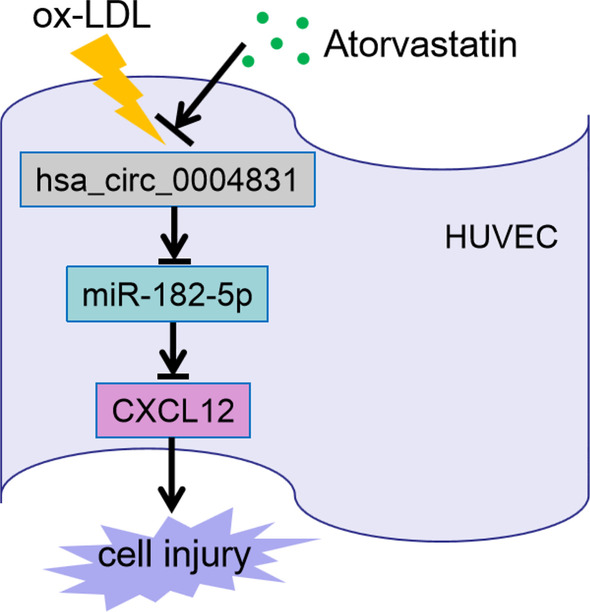
The scheme displayed the main design of this study

Here, we constructed the pathologic conditions of AS in HUVECs by treating ox-LDL and investigated the effects of ATV on HUVEC viability, apoptosis, cycle, oxidative stress and inflammatory response. Besides, we determined the expression of a novel circRNA (hsa_circ_0004831) in ox-LDL-administered HUVECs with or without ATV treatment, and we performed gain- and loss-function assays of hsa_circ_0004831 to reveal its function. In addition, the functional mechanism of hsa_circ_0004831 in ATV-prevented HUVEC injuries associated with miR-482-5p and CXCL12 was explored (Fig. 1). Our study aimed to provide evidence that hsa_circ_0004831 acted as an ATV-responded biomarker in AS treatment.

## Methods

### HUVEC treatment

HUVECs provided by Procell Co., Ltd., (Wuhan, China) were maintained in specific medium (Ham’s F-12K + 0.1 mg/mL heparin + 0.05 mg/mL endothelial cell growth supplement + 10% fetal bovine serum) (Procell Co., Ltd.,) at a 37℃ incubator containing 5% CO_2_.

Ox-LDL and ATV were purchased from Yeasen (Shanghai, China) and Solarbio (Beijing, China), respectively. To construct endothelial cell injury models for AS, HUVECs were treated with ox-LDL at various doses (0, 25, 50, 100 and 200 mg/L). ATV was dissolved in ethanol (20 mg/mL), and ox-LDL-treated HUVECs were administered with different concentrations of ATV (0, 2.5, 5 and 10 μM) for 24 h. Following experiments were performed using ox-LDL at 100 mg/L and using ATV at 10 μM.

### CCK-8 assay

To detect cell viability, HUVECs with different treatments or transfection were seeded into 96-well plates (5 × 10^3^ cells/well) and cultured for 24 h. Then, 10 μL CCK-8 reagent (Solarbio Co., Ltd.) was added into each well. After 2 h, the absorbance at 450 nm was detected to obtain cell viability.

### Quantitative real-time PCR (qPCR)

Total RNA was obtained from cells by treating Trizol reagent (Invitrogen, Carlsbad, CA, USA). For reverse transcription analysis, 1 μg RNA was utilized to assemble cDNA using mirVana™ qPCR miRNA Detection Kit (Invitrogen) or using First Strand cDNA Synthesis Kit (Thermo Fisher Scientific, Waltham, MA, USA). Then, diluted cDNA was used for qPCR reaction using SYBR Green qPCR Kit (Thermo Fisher Scientific) in line with the guidelines. GAPDH or U6 was used as the internal reference, using the 2^−ΔΔCt^ method for calculation. The primers used were: hsa_circ_0004831 (F: 5′-AGAAGAAAGAGCGTGCCGAA-3′ and R: 5′-TGATCATCAGAGGAGGGCGA-3′); RSF1 (F: 5′-GCAAGGTAAAACCCAAAGGCA-3′ and R: 5′-TTCACTGCCAGACCCTTCAC-3′); miR-182-5p (F: 5′-GCGTTTGGCAATGGTAGAACT-3′ and R: 5′-AGTGCAGGGTCCGAGGTATT-3′); CXCL12 (F: 5′-TGCCCTTCAGATTGTAGCCC-3′ and R: 5′-CTGTAAGGGTTCCTCAGGCG-3′); GAPDH (F: 5′-CATGGGTGTGAACCATGAGAAGTA-3′ and R: 5′-CAGTAGAGGCAGGGATGATGTTCT-3′); U6 (F: 5′-CTCGCTTCGGCAGCACA-3′ and R: 5′-AACGCTTCACGAATTTGCGT-3′);

### RNase R and actinomycin D treatment

RNA extracts were administered with 3 U/μg RNase R (Epicentre, Madison, WI, USA) for 15 min at 37℃. Then, qPCR was used to ascertain hsa_circ_0004831 expression and RSF1 expression. HUVECs in 96-well plates were exposed to 2 μg/mL actinomycin D (Sigma-Aldrich, St. Louis, MO, USA) for 0, 4, 8, 12 and 24 h. Then, cells were collected for RNA isolation and qPCR analysis.

### Oligonucleotides, plasmids and cell transfection

SiRNA was used for hsa_circ_0004831 knockdown (si-circRNA#1 and si-circRNA#2) or CXCL12 knockdown (si-CXCL12#1 and si-CXCL12#2), which were synthesized by Ribobio (Guangzhou, China), with si-NC as negative control for them. For hsa_circ_0004831 overexpression, hsa_circ_0004831 was cloned into pCD-ciR plasmid (circRNA) by Geneseed (Guangzhou, China), with pCD-ciR empty plasmid (pCD-ciR) as negative control. For miR-182-5p overexpression or inhibition, miR-182-5p mimic (miR-182-5p) and miR-182-5p inhibitor (anti-miR-182-5p) were purchased from Ribobio, with miR-NC or anti-miR-NC as negative control. HUVECs were used for transfection using the Lipofectamine 3000 transfection kit (Invitrogen).

### Flow cytometry assay

Annexin V-FITC Apoptosis Detection Kit (Invitrogen) was applied in this assay. Simply put, cells were washed with PBS and resuspended in 200 μL Binding Buffer at a density of 3 × 10^5^ cells/mL, followed by treatment with 5 μL Annexin V-FITC and 10 µL Propidium Iodide (PI). Cell apoptosis was examined using a FACS cytometer (BD Biosciences, San Jose, CA, USA).

Flow cytometry assay of cell cycle was performed using Tali™ Cell Cycle Kit (Invitrogen). In brief, 70% ethanol was then used to fix cells at -20℃ overnight. Subsequently, cells (3 × 10^4^) were stained with the cell cycle solution (containing PI and RNase A) after washing with PBS. Cell distribution was ascertained using a FACS cytometer (BD Biosciences).

### The detection of oxidative stress

The levels of oxidative stress markers, including MDA and SOD, were measured to monitor the status of oxidative stress using the MDA assay kit (Beyotime, Shanghai, China) and SOD assay kit (Beyotime), respectively.

### The detection of inflammatory response

The release of proinflammatory factors, including IL-6 and IL-1β, was detected to monitor inflammatory response using human IL-6 ELISA kit (Beyotime) and human IL-1β ELISA kit (Beyotime), respectively.

### Bioinformatics analysis

Bioinformatics tools, including circinteractome (https://circinteractome.nia.nih.gov/) and starbase (http://starbase.sysu.edu.cn/), were applied in this study.

### Biotin RNA pull-down assay

Biotin-labeled probes targeting hsa_circ_0004831 (Bio-circRNA) or miR-182-5p (Bio-miR-182-5p) were obtained from Ribobio, with Bio-NC as the corresponding negative control. HUVECs were transfected with above mentioned biotin-labeled probes, and then RNA pull-down assay was carried out using Pierce™ Magnetic RNA–Protein Pull-Down Kit (Pierce, Rockford, IL, USA) as early described [16].

### Subcellular distribution

Cellular fractionation was conducted using the PARIS Kit (Ambion, Austin, TX, USA) to isolate cytoplasmic and nuclear RNA. Cytoplasmic RNA and nuclear RNA were then subjected to qPCR to ascertain the expression of hsa_circ_0004831, using GAPDH and U6 as the internal references in the cytoplasm or nucleus, respectively.

### Dual-luciferase reporter assay

Hsa_circ_0004831 sequence fragment or CXCL12 3′UTR sequence fragment containing wild or mutant miR-182-5p binding sites was amplified and inserted into pmiRGLO plasmid (Promega, Madison, WI, USA), naming as wt-hsa_circ_0004831, mut-hsa_circ_0004831, wt-CXCL12 3′UTR and mut-CXCL12 3′UTR. HUVECs were planted into 6-well plates with the transfection of miR-182-5p or miR-NC and wt-hsa_circ_0004831, mut-hsa_circ_0004831, wt-CXCL12 3′UTR and mut-CXCL12 3′UTR, respectively, followed by the detection of luciferase activity using the dual-luciferase reporter assay system (Promega).

### RIP

RIP was implemented using a Magna RIP Kit (Millipore, Billerica, MA, USA) following the protocols. The beads were coated with human Ago2 antibody (anti-Ago2; Millipore) or mouse IgG antibody (anti-IgG; Millipore; control). Cells were lysed and cell lysates were incubated with anti-Ago2- or anti-IgG-coated beads. Afterwards, the coprecipitated RNAs were extracted and detected.

### Western blot

Total proteins were extracted using RIPA buffer (Solarbio), separated by 12% SDS-PAGE gels and then electro-transferred to PVDF membranes (Millipore). The membranes containing proteins were blocked using 5% skim milk, followed by the incubation of the primary antibodies against CXCL12 (ab18919; Abcam, Cambridge, MA, USA) and GAPDH (ab8245; Abcam). Next, the membranes containing proteins were incubated with the secondary antibody (ab205719; Abcam). Protein blots were finally shown using the ECL kit (Beyotime) and quantified by Image J software (NIH, Bethesda, MA, USA).

### Statistical analysis

Statistical analysis was conducted using GraphPad Prism 7 (GraphPad; La Jolla, CA, USA). Statistical significance was assessed using Student’s *t*-test or the analysis of variance (ANOVA) test between two groups or among multiple groups. All data were obtained from three independent repetitions and then displayed as mean ± standard deviation (SD). *P* < 0.05 represented statistical significance.

## Result

### Hsa_circ_0004831 was highly expressed in ox-LDL-treated HUVECs, while its expression was declined with the addition of ATV

**Fig. 2:**
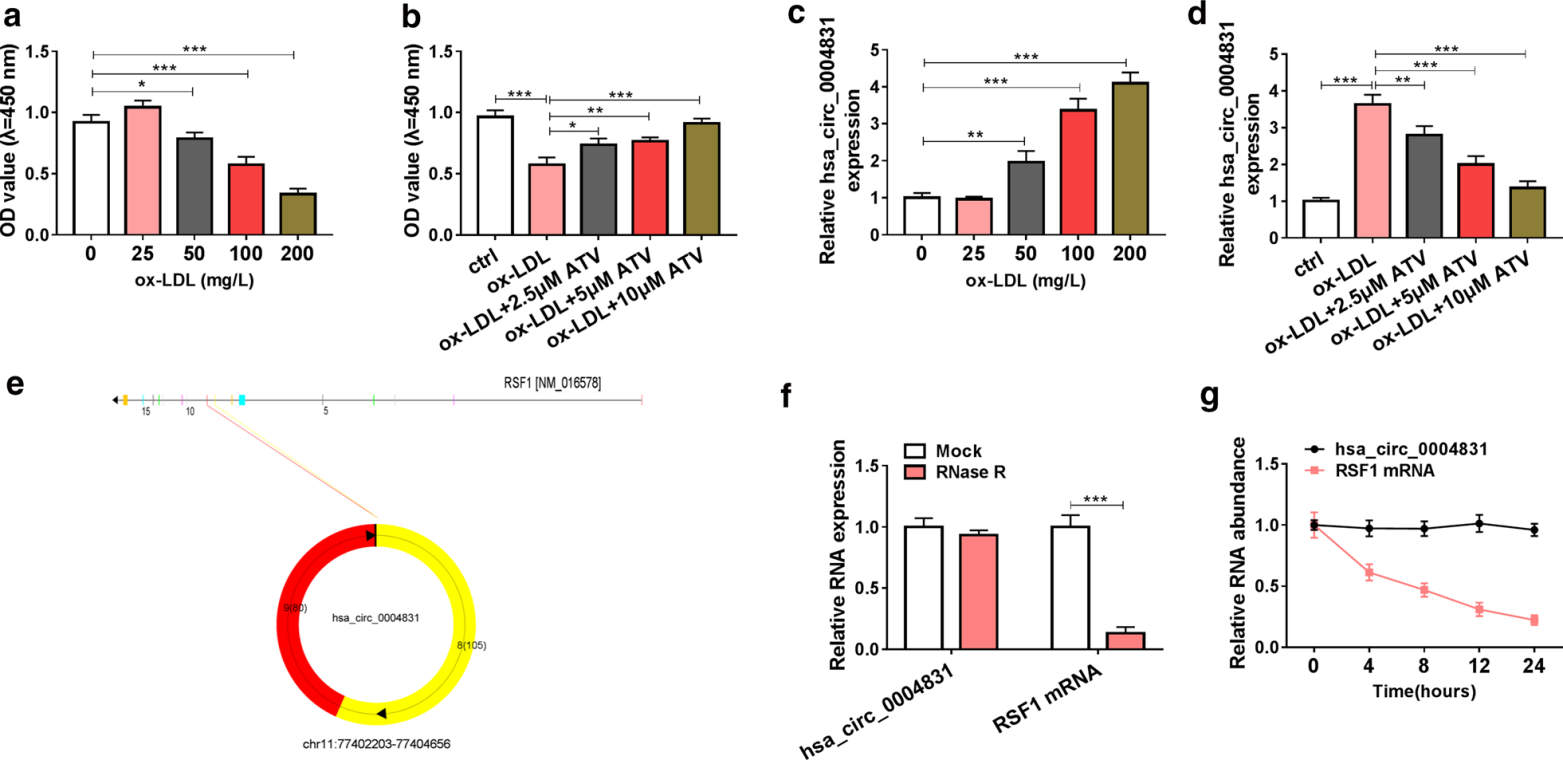
The expression of hsa_circ_0004831 was promoted in ox-LDL-treated HUVECs but impaired in ox-LDL-treated HUVECs with the addition of ATV. **a** The treatment of different concentrations of ox-LDL (0, 25, 50, 100 and 200 mg/L) on HUVEC viability. **b** Cell viability in 100 mg/L ox-LDL-treated HUVECs with the addition of different concentrations of ATV (0, 2.5, 5 and 10 μM). **c** The expression of hsa_circ_0004831 in ox-LDL-treated HUVECs. **d** The expression of hsa_circ_0004831 in ox-LDL-treated HUVECs with the addition of ATV. **e** Schematic diagram revealed the formation of hsa_circ_0004831. **f**, **g** The stability of hsa_circ_0004831 was examined by RNase R and actinomycin D. *P < 0.05, **P < 0.01, ***P < 0.001

The cell model of AS was constructed in HUVECs treated with ox-LDL. As a result, ox-LDL treatment led to a notable decrease of cell viability in a dose-dependent manner (Fig. 2a). However, the addition of ATV significantly promoted ox-LDL-depleted cell viability (Fig. 2b). The data suggested that ATV recovered cell viability that was weakened by ox-LDL. Interestingly, we found that hsa_circ_0004831 expression was gradually promoted with the increase of ox-LDL concentration in HUVECs (Fig. 2c), while the expression of hsa_circ_0004831 was notably lessened in ox-LDL-treated HUVECs with the addition of ATV (Fig. 2d). Structure analysis showed that hsa_circ_0004831 was derived from the exon8-9 of RSF (NM_016578), with 185 bp in length (Fig. 2e). Then, we tested the stability of hsa_circ_0004831 using RNase R and actinomycin D. we found that the expression of hsa_circ_0004831 was not affected, while the expression of RSF1 mRNA was strikingly reduced by RNase R treatment (Fig. 2f). Besides, actinomycin D resulted in a decrease of RSF1 mRNA expression but not hsa_circ_0004831 expression (Fig. 2g). The data indicated that hsa_circ_0004831 was involved in ox-LDL-induced HUVEC injury.

### ATV rescued ox-LDL-induced HUVEC injury by decreasing hsa_circ_0004831 expression

**Fig. 3:**
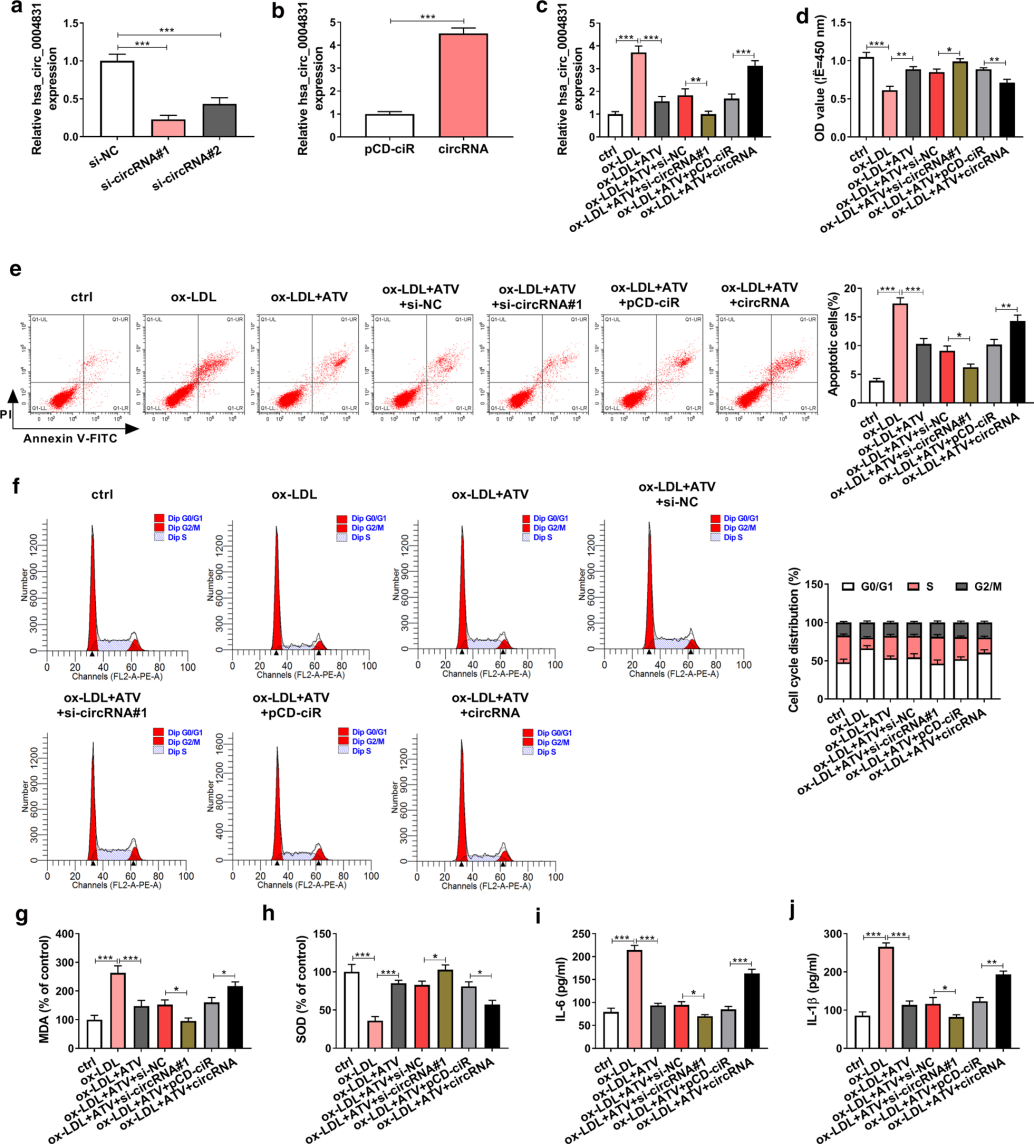
ATV alleviated ox-LDL-induced HUVEC injuries by suppressing the expression of hsa_circ_0004831. **a**, **b** The efficiency of hsa_circ_0004831 interference or hsa_circ_0004831 overexpression. Ox-LDL-treated HUVECs with the addition of ATV were transfected with si-circRNA#1 or circRNA, with si-NC or pCD-ciR as the control. Then, **c** the expression of hsa_circ_0004831 in these groups. **d** Cell viability was checked using CCK-8 assay. **e**, **f** Cell apoptosis and cell cycle were analyzed in these cells using flow cytometry assay. **g**, **h** The levels of MDA and SOD. **i**, **j** The release of IL-6 and IL-1β. *P < 0.05, **P < 0.01, ***P < 0.001

Gain- or loss-function experiments of hsa_circ_0004831 were performed to determine the role of hsa_circ_0004831 in endothelial injury. The efficiency of hsa_circ_0004831 knockdown or overexpression was checked at first, and we found the expression of hsa_circ_0004831 was remarkably declined in HUVECs transfected with si-circRNA#1 or si-circRNA#2 compared to si-NC (Fig. 3a). The expression of hsa_circ_0004831 was remarkably reinforced in HUVECs transfected with circRNA compared to pCD-ciR (Fig. 3b). The expression of hsa_circ_0004831 was further reduced in ox-LDL + ATV-treated HUVECs transfected with si-circRNA#1 relative to si-NC, while hsa_circ_0004831 expression was notably reinforced in ox-LDL + ATV-treated HUVECs transfected with circRNA relative to pCD-ciR (Fig. 3c). In function, ATV recovered ox-LDL-suppressed cell viability, and hsa_circ_0004831 knockdown further promoted cell viability, while hsa_circ_0004831 overexpression partly weakened cell viability (Fig. 3d). Besides, ox-LDL-induced cell apoptosis and cell cycle arrest were attenuated by ATV, and hsa_circ_0004831 knockdown further inhibited cell apoptosis and cell cycle arrest, while hsa_circ_0004831 overexpression promoted cell apoptosis and cell cycle arrest (Fig. 3e, f). As for oxidative stress, the level of MDA was enhanced by ox-LDL but repressed by the addition of ATV, and hsa_circ_0004831 knockdown further suppressed their levels, while hsa_circ_0004831 overexpression reversely promoted the level of MDA (Fig. 3g). The level of SOD in these experimental groups was opposite to the level of MDA (Fig. 3h). As for inflammatory response, the release of IL-6 and IL-1β induced by ox-LDL was suppressed by the addition of ATV, and hsa_circ_0004831 knockdown further reduced the release of these factors, while hsa_circ_0004831 overexpression partly recovered their release (Fig. 3i, j). These findings displayed that ATV alleviated ox-LDL-induced HUVEC injuries, including apoptosis, cell cycle arrest, oxidative stress and inflammation, by decreasing the expression of hsa_circ_0004831.

### MiR-182-5p was a target of hsa_circ_0004831

**Fig. 4:**
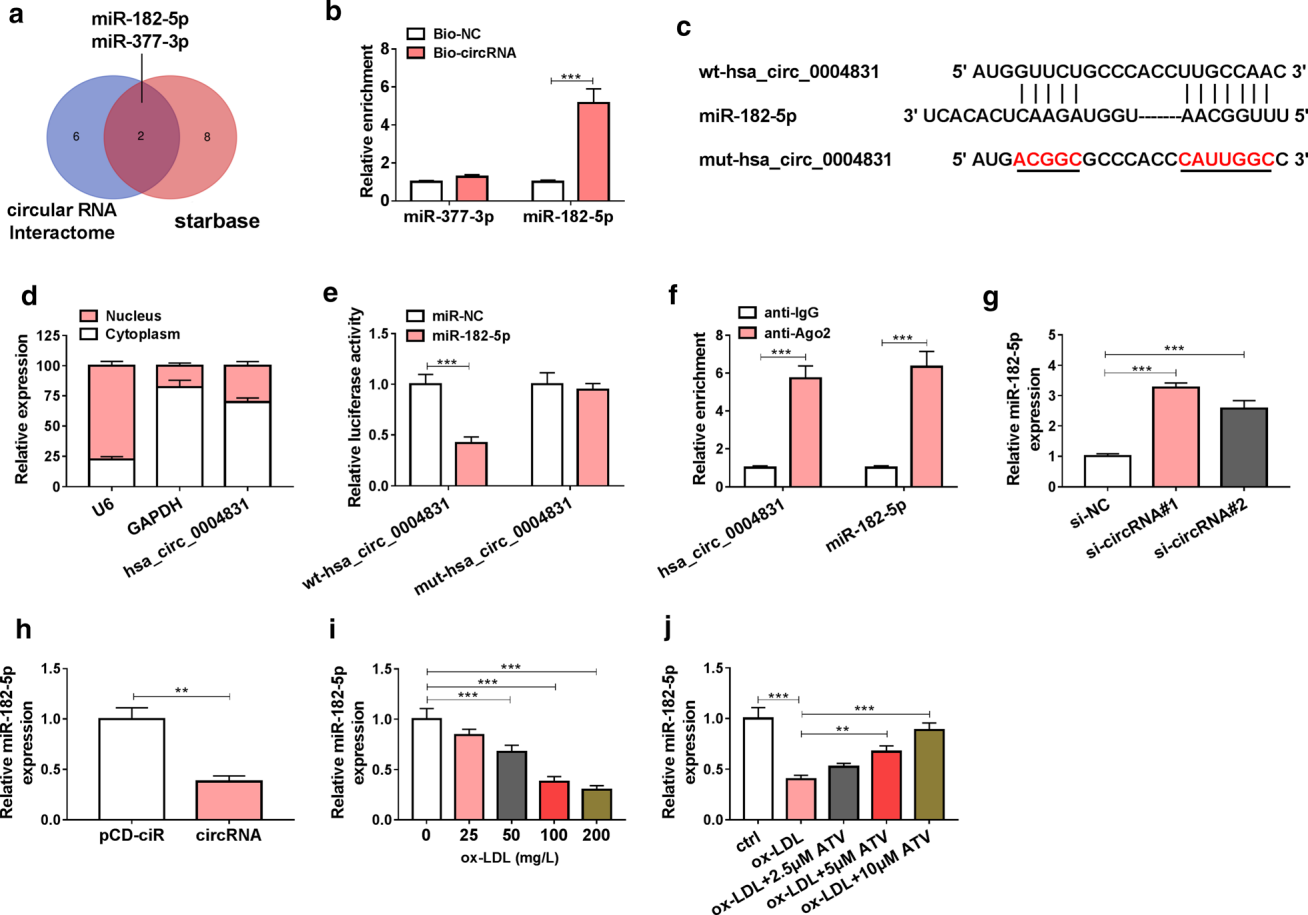
MiR-182-5p was a target of hsa_circ_0004831. **a** MiR-182-5p and miR-377-3p were predicted as targets of hsa_circ_0004831 both by circular RNA interactome and starbase. **b** Biotin RNA pull-down assay was performed to screen miR-182-5p and miR-377-3p. **c** The binding sites between hsa_circ_0004831 and miR-182-5p. **d** The distribution of hsa_circ_0004831 in the cytoplasm or nucleus was determined by subcellular distribution assay. **e**, **f** The relationship between hsa_circ_0004831 and miR-182-5p was validated by dual-luciferase reporter assay and RIP assay. **g**, **h** The effect of hsa_circ_0004831 knockdown or overexpression on the expression of miR-182-5p. **i** The expression of miR-182-5p in HUVECs treated with different concentrations of ox-LDL. **j** The expression of miR-182-5p in ox-LDL-treated HUVECs with the addition of ATV. **P < 0.01, ***P < 0.001

We speculated that hsa_circ_0004831 might be involved in HUVEC injury by targeting miRNAs. To verify this hypothesis, we identified the miRNAs targeted by hsa_circ_0004831. The data from circular RNA Interactome and starbase predicted that miR-182-5p and miR-377-3p were targets of hsa_circ_0004831 (Fig. 4a). Then, biotin-labeled RNA pull-down assay showed that Bio-circRNA significantly enriched the abundance of miR-182-5p but not miR-377-3p (Fig. 4b). Hsa_circ_0004831 harbored special binding sites in miR-182-5p sequence, then the mutations in these sites were designed for the following experiments (Fig. 4c). Subsequent assay showed that hsa_circ_0004831 was mainly distributed in the cytoplasm compared to the nucleus (Fig. 4d), which provided a basis for hsa_circ_0004831 acting as a molecular sponge of miRNAs. Luciferase activity was markedly decreased in HUVECs transfected with wt-hsa_circ_0004831 and miR-182-5p compared to miR-NC (Fig. 4e). RIP assay suggested that both hsa_circ_0004831 and miR-182-5p were notably enriched in the anti-Ago2 group compared with anti-IgG group (Fig. 4f). Moreover, the expression of miR-182-5p was promoted in HUVECs transfected with si-circRNA#1 or si-circRNA#2, while its expression was impaired in HUVECs transfected with circRNA compared to pCD-ciR (Fig. 4g, h). In addition, the expression of miR-182-5p was notably impaired in ox-LDL-treated HUVECs (Fig. 4i), while the addition of ATV recovered the expression of miR-182-5p (Fig. 4j). All data demonstrated that miR-182-5p was a target of hsa_circ_0004831.

### MiR-182-5p restoration could partly abolish the effects of hsa_circ_0004831 overexpression-induced HUVEC injuries

**Fig. 5:**
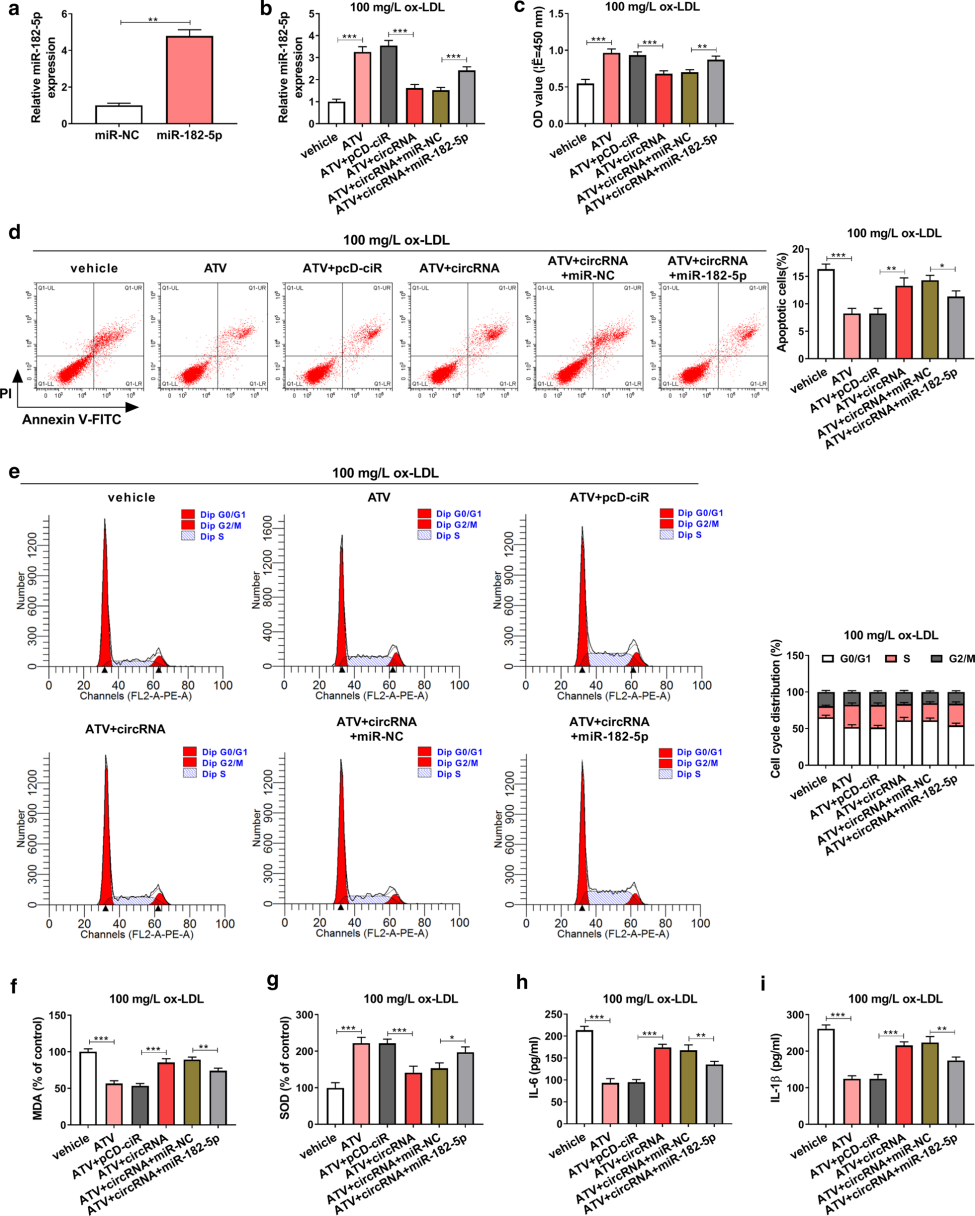
Hsa_circ_0004831 overexpression reversed the effect of ATV by targeting miR-182-5p in ox-LDL-treated HUVECs. **a** The efficiency of miR-182-5p mimic. 100 mg/L ox-LDL-treated HUVECs with the addition of ATV were transfected with circRNA or circRNA + miR-182-5p, with pCD-ciR or miR-NC as the control. **b** The expression of miR-182-5p in these cells was checked by qPCR. **c** Cell viability in these cells was checked using CCK-8 assay. **d**, **e** Cell apoptosis and cell cycle in these cells were monitored using flow cytometry assay. **f**, **g** The levels of MDA and SOD in these cells. **h**, **i** The release of IL-6 and IL-1β in these cells. *P < 0.05, **P < 0.01, ***P < 0.001

The efficiency of miR-182-5p mimic was checked, and the expression of miR-182-5p was significantly upregulated in HUVECs transfected with miR-182-5p compared to miR-NC (Fig. 5a). Then, the expression of miR-182-5p induced in ox-LDL (100 mg/L)-treated HUVECs treated with ATV was impaired by the transfection of circRNA, while miR-182-5p expression was recovered by the reintroduction of miR-182-5p (Fig. 5b). In HUVECs treated with ox-LDL and ATV, miR-182-5p reintroduction recovered cell viability that was impaired by circRNA transfection alone (Fig. 5c). Besides, miR-182-5p reintroduction alleviated cell apoptosis and cell cycle arrest were promoted by hsa_circ_0004831 overexpression alone in HUVECs treated with ox-LDL and ATV (Fig. 5d, e). In addition, the level of MDA was further enhanced in ox-LDL and ATV-treated HUVECs with hsa_circ_0004831 overexpression but partly lessened in ox-LDL and ATV-treated HUVECs with hsa_circ_0004831 overexpression and miR-182-5p enrichment, while the level of SOD in these groups was opposite to the level of MDA (Fig. 5f, g). Moreover, the release of IL-6 and IL-1β enhanced in ox-LDL and ATV-treated HUVECs transfected with circRNA was largely alleviated in ox-LDL and ATV-treated HUVECs transfected with circRNA + miR-182-5p (Fig. 5h, i). These data indicated that hsa_circ_0004831 was involved in HUVEC injuries by targeting miR-182-5p.

### MiR-182-5p bound to CXCL12

**Fig. 6:**
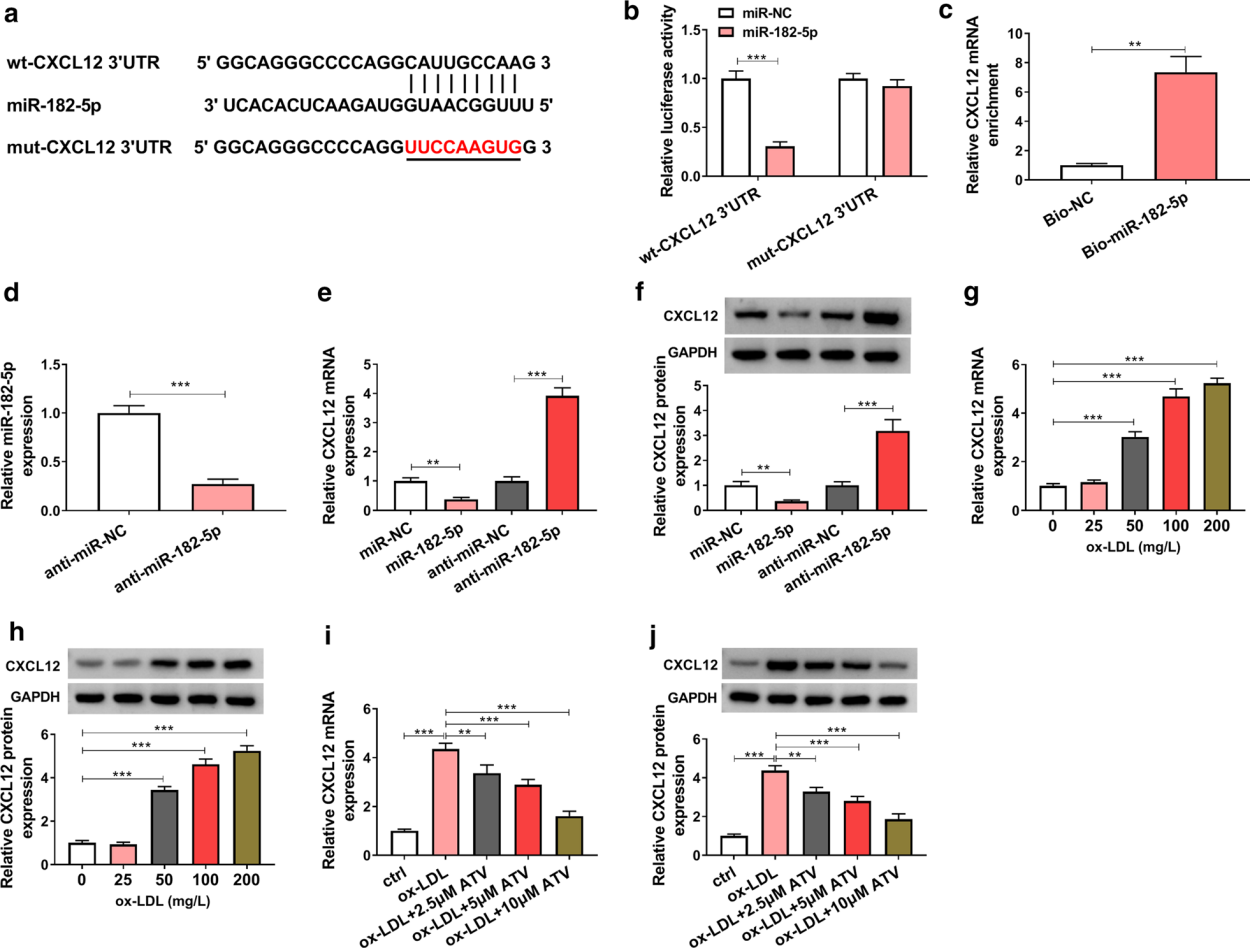
CXCL12 was a target of miR-182-5p. **a** The binding sites between miR-182-5p and CXCL12 3′UTR were analyzed by starbase. **b**, **c** The interaction between miR-182-5p and CXCL12 was verified by dual-luciferase reporter assay and biotin RNA pull-down assay. **d** The efficiency of miR-182-5p inhibitor was checked using qPCR. **e**, **f** The expression of CXCL12 in HUVECs with miR-182-5p overexpression or inhibition was detected using qPCR and western blot. **g**, **h** The expression of CXCL12 in ox-LDL-treated HUVECs was detected by qPCR and western blot. **i**, **j** The expression of CXCL12 in ox-LDL-treated HUVECs with the addition of ATV was detected by qPCR and western blot. **P < 0.01, ***P < 0.001

Regarding that miRNAs play functions by binding to mRNA 3′UTR. Following this mechanism, we identified the potential mRNAs targeted by miR-182-5p. The analysis from starbase predicted that miR-182-5p bound to CXCL12 3′UTR via a special binding site (Fig. 6a). Besides, the cotransfection of miR-182-5p and wt-CXCL12 3′UTR notably reduced the luciferase activity in HUVECs, while the co-transfection of miR-182-5p and mut-CXCL12 3′UTR hardly affected the luciferase activity (Fig. 6b). Biotin RNA pull-down assay showed that Bio-miR-182-5p could enrich a high abundance of CXCL12 (Fig. 6c). These assays verified that CXCL12 was a target of miR-182-5p. The efficiency of miR-182-5p inhibitor was examined, and we found that the expression of miR-182-5p was strikingly declined in HUVECs transfected with anti-miR-182-5p compared to anti-miR-NC (Fig. 6d). The expression of CXCL12 was significantly decreased in miR-182-5p-overexpressed cells, while CXCL12 expression was significantly enhanced in miR-182-5p-downregulated cells at both mRNA and protein levels (Fig. 6e, f). Moreover, the expression of CXCL12 was markedly strengthened in ox-LDL-treated HUVECs in a dose-dependent manner (Fig. 6g, h), while the expression of CXCL12 was gradually impaired with the addition of ATV (Fig. 6i, j). These data suggested that miR-182-5p suppressed CXCL12 expression by binding to CXCL12 3′UTR.

### MiR-182-5p inhibition recovered ATC-suppressed HUVEC injuries by enriching the level of CXCL12

**Fig. 7:**
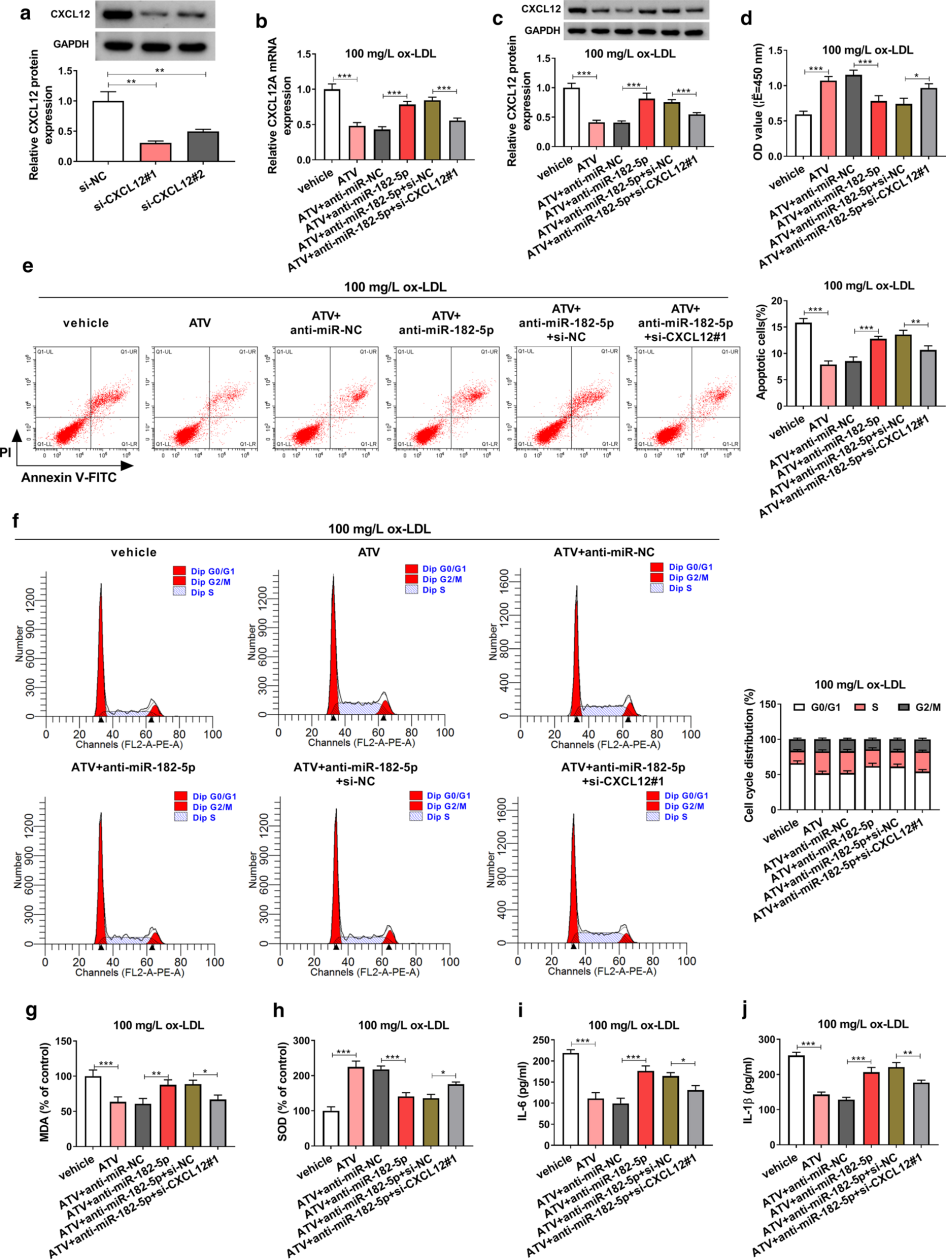
MiR-182-5p inhibition reversed the effect of ATV by increasing the expression of CXCL12 in ox-LDL-treated HUVECs. **a** The efficiency of CXCL12 interference. 100 mg/L ox-LDL-treated HUVECs with the addition of ATV were transfected with anti-miR-182-5p or anti-miR-182-5p + si-CXCL12#1, with anti-miR-NC or anti-miR-182-5p + si-NC. **b**, **c** The expression of CXCL12 mRNA and protein in these cells. **d** Cell viability in these cells. **e**, **f** Cell apoptosis and cell cycle were investigated using flow cytometry assay. **g**, **h** The levels of MDA and SOD in these cells. **i**, **j** The release of IL-6 and IL-1β in these cells *P < 0.05, **P < 0.01, ***P < 0.001

Given that CXCL12 was a target of miR-182-5p, we speculated that miR-182-5p exerted its role by degrading CXCL12. Rescue experiments were then performed to clarify this hypothesis. The efficiency of CXCL12 interference was checked, and the data from western blot showed that CXCL12 expression level was strikingly decreased in HUVECs transfected with either si-CXCL12#1 or si-CXCL12#2, and the efficiency of si-CXCL12#1 was higher (Fig. 7a). In ox-LDL-treated HUVECs with the addition of ATV, the expression of CXCL12 at both mRNA and protein levels was notably promoted by the transfection of anti-miR-182-5p alone but impaired by the transfection of anti-miR-182-5p + si-CXCL12#1 (Fig. 7b, c). In function, ATV-rescued cell viability was impaired by miR-182-5p inhibition, while further CXCL12 interference recovered cell viability (Fig. 7d). ATV-blocked cell apoptosis and cell cycle arrest were notably promoted by the transfection of anti-miR-182-5p, while the transfection of anti-miR-182-5p + si-CXCL12#1 partly lessened cell apoptosis and cell cycle arrest (Fig. 7e, f). For oxidative stress, the level of MDA impaired in ATV-supplemented HUVECs was restored by anti-miR-182-5p transfection but further weakened by anti-miR-182-5p + si-CXCL12#1 transfection, while the level of SOD in these experiments groups was opposite to MDA level (Fig. 7g, h). For inflammatory response, the release of IL-6 and IL-1β blocked by ATV treatment was stimulated by miR-182-5p inhibition, while further CXCL12 interference partly repressed the release of these factors (Fig. 7i, j). The data indicated that miR-182-5p responded to ATV-alleviated HUVEC injuries by regulating the expression of CXCL12.

### Hsa_circ_0004831 overexpression promoted CXCL12 expression by targeting miR-182-5p

**Fig. 8:**
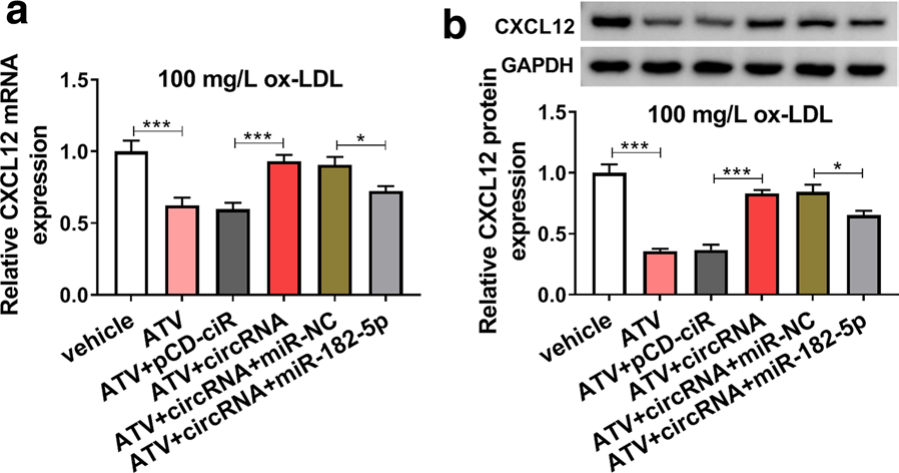
Hsa_circ_0004831 overexpression promoted the expression of CXCL12 by targeting miR-182-5p. 100 mg/L ox-LDL-treated HUVECs with the addition of ATV were transfected with circRNA or circRNA + miR-182-5p, with pCD-ciR or circRNA + miR-NC as the control. **a**, **b** The expression of CXCL12 at both mRNA and protein levels in these cells. *P < 0.05, ***P < 0.001

Furthermore, we found the expression of CXCL12 impaired in ATV-treated HUVECs containing ox-LDL was significantly promoted by the transfection of circRNA compared to pCD-ciR, while CXCL12 expression was partly depleted by the transfection of circRNA + miR-182-5p at both mRNA and protein levels (Fig. 8a, b). The data suggested that hsa_circ_0004831 promoted CXCL12 expression by targeting miR-182-5p in the pathological environment of ATV-blocked endothelial injury.

## Discussion

Our present study monitored that ox-LDL induced HUVEC apoptosis, oxidative stress and inflammation, while ATV attenuated these injured effects. Interestingly, we observed that hsa_circ_0004831 expression was markedly elevated by ox-LDL administration but repressed by additional ATV treatment in HUVECs. Further analysis disclosed that ATV agent alleviated ox-LDL-triggered HUVEC apoptosis, oxidative stress and inflammation by regulating the hsa_circ_0004831/miR-182-5p/CXCL12 regulatory network.

ATV is a competitive inhibitor of 3-hydroxy-3-methylglutaryl-coenzyme A reductase, suppressing the synthesis of cholesterol [17]. Previous studies demonstrated that ATV inhibited AS progression through multiple mechanisms. For example, ATV depleted the levels of ipoprotein remnants generated by activated brown fat, thus playing anti-atherogenic effects [18]. Besides, ATV weakened the extent of local inflammation in atherosclerotic plaques to combat AS [19]. Moreover, ATV treatment blocked the apoptotic rate and the migration ability in ox-LDL-administered HUVECs [20]. ATV usage regulated the expression of inflammatory and oxidant markers (the decrease of C-reactive protein and Myeloperoxidase, and the increase of SOD) in plasma samples of AS patients [21]. In agreement with these opinions, our data observed that ATV repressed cell apoptosis, lessened oxidative by reducing MDA level and enhancing SOD level, and alleviated inflammatory responses by decreasing the release of IL-6 and IL-1β in ox-LDL-administered HUVECs. The data showed that ATV prevented AS progression partly by suppressing ox-LDL-induced HUVEC dysfunctions and injuries, which strongly supported that ATV was an effective anti-atherosclerosis agent in clinical practice.

Interestingly, we found that hsa_circ_0004831 expression was enhanced in ox-LDL-administered HUVECs but impaired by the addition of ATV. In function, hsa_circ_0004831 overexpression partly abolished the therapeutic effects of ATV, thereby restoring ox-LDL-induced apoptosis, oxidative stress and inflammation in HUVECs, while hsa_circ_0004831 knockdown played the opposite role. Hsa_circ_0004831 was also shown to be overexpressed in ox-LDL-treated HUVECs [22]. However, there was no evidence supporting the role of hsa_circ_0004831 in AS or in ox-LDL-treated HUVECs. Our study for the first time explored the function of hsa_circ_0004831 in ox-LDL-administered HUVECs, revealing that ATV played anti-atherosclerosis effects by reducing the expression of hsa_circ_0004831. The inhibition of hsa_circ_0004831 might be a promising strategy for the treatment of AS.

Further mechanism exploration presented that miR-182-5p was targeted by hsa_circ_0004831. A previous study disclosed the role of miR-182-5p in AS models and clarified that miR-182-5p was downregulated by ox-LDL in mouse macrophages, and miR-182-5p restoration inhibited the formation of foam cells, apoptosis and oxidative stress in ox-LDL-treated mouse macrophages by degrading toll-like receptor 4 (TLR4) [23]. Vascular calcification was prevalent in patients with AS, and miR-182-5p was also shown to be downregulated in vascular calcification mouse models and β-glycerophosphoric-induced calcified vascular smooth muscle cells [24]. These findings highlighted the anti-atherosclerosis role of miR-182-5p. Consistent with these findings, miR-182-5p expression was also declined by ox-LDL treatment in HUVECs but recovered by the addition of ATV in our study. In function, miR-182-5p overexpression reversed the promoted effects on apoptosis, oxidative stress and inflammation caused by hsa_circ_0004831 overexpression, while miR-182-5p inhibition also impaired the therapeutic effects of ATV in ox-LDL-treated HUVECs.

Furthermore, CXCL12 was confirmed to be a target of miR-182-5p, and hsa_circ_0004831 upregulated the expression of CXCL12 by suppressing miR-182-5p. Thus, the regulatory axis of hsa_circ_0004831/miR-182-5p/CXCL12 was generated to reveal the mechanism of hsa_circ_0004831 responding to ATV. CXCL12 was closely associated with AS progression, and elevated expression of CXCL12 was involved in various AS pathological processes, such as dyslipidemia, inflammation, neointima hyperplasia and angiogenesis [25]. CXCL12 synergistically promoted the ox-LDL-induced oxidative stress, thrombogenic impact on platelet function [26]. Consistent with their data, we found that CXCL12 knockdown eliminated the promoted effects on apoptosis, oxidative stress and inflammation caused by miR-182-5p inhibition, thus playing a proatherogenic role in AS progression.

## Conclusion

In conclusion, ATV agent alleviated ox-LDL-induced apoptosis, oxidative stress and inflammatory response in HUVECs. Hsa_circ_0004831 was upregulated in ox-LDL-treated HUVECs but reduced by the addition of ATV. Hsa_circ_0004831 responded to ATV therapeutic effects on ox-LDL-induced HUVEC dysfunctions and injuries by regulating the miR-182-5p/CXCL12 axis. Our study strongly supports that ATV inhibits HUVEC injuries by mediating hsa_circ_0004831.

## Data Availability

All data generated or analysed during this study are included in this published article.
